# Numerical Design of Plasmonic Structure Integrated Superconducting Nanowire Single-Photon Detector Constructed with BSCCO Patterns

**DOI:** 10.3390/mi17080887

**Published:** 2026-07-25

**Authors:** András Szenes, László Pothorcki, Balázs Bánhelyi, Mária Csete

**Affiliations:** 1 Department of Optics and Quantum Electronics, University of Szeged, Dóm tér 9, 6720 Szeged, Hungary; 2 HUN-REN Wigner Research Center of Physics, Konkoly-Thege Miklós út 29-33, 1121 Budapest, Hungary; 3 Department of Computational Optimization, University of Szeged, Árpád tér 2, 6720 Szeged, Hungary

**Keywords:** single-photon detector, numerical optimization, plasmonics, nanocavity, cuprate, effective medium

## Abstract

Superconducting nanowire single-photon detectors (SNSPDs) were numerically designed by integrating periodic plasmonic structures onto hBN-protected superconducting BSCCO patterns to enhance absorptance. The numerical investigation of optimized nanocavity array (NCAI) and nanocavity trench array (NCTAI) SNSPDs has revealed that more than one-order-of-magnitude higher absorptance can be achieved at perpendicular incidence, compared to the corresponding meandered BSCCO pattern in a simple resonant optical cavity. The presented SNSPD designs are considerably improved via first and third quarter wavelength nanocavity resonances, as evidenced by the near-field maps and validated by the standard retrieval method. Although the NCAI-SNSPD exhibits a slightly larger absorptance, the NCTAI-SNSPD remains potentially competitive due to its smaller filling factor, which may be beneficial for reduced charge-crowding effect.

## 1. Introduction

The detection of single photons encompasses numerous fields of fundamental research and applications, including secure information transfer, and plays a crucial role in quantum computing and quantum communication [1,2,3]. Among state-of-the-art single-photon detectors, superconducting nanowire single-photon detectors (SNSPDs) have remarkable properties [4]. These detectors operate based on the modification of the superconducting state in the nanowires constituting their absorbing segments, as the single-photon-induced hotspot is capable of breaking the superconducting equilibrium. The consequent increase beyond the critical current manifests itself in a voltage jump in the readout electronics [5]. Exploiting this phenomenon, SNSPDs offer operation across a wide range of wavelengths (from X-ray to mid-IR) and enable high efficiency as well as picosecond time-resolution [6,7,8,9]. A drawback of SNSPDs is that their operation is restricted to very low temperatures, typically below 4.2 K, in the case of, e.g., niobium-nitride (NbN) superconductors. This cryogenic temperature requirement constrains their broad applicability and increases the overall system cost.

Efforts have been made to develop high-critical-temperature cuprate superconductors for SNSPDs, yet experimentally detecting single photons above 4.2 K was not successful [10,11]. The primary challenge is their manufacturing, as traditional SNSPD fabrication procedures severely damage cuprate superconductors. Recently, a method was developed whereby non-superconducting patterns are drawn into helium-ion-exposed Bi_2_Sr_2_CaCu_2_O_8+δ_ (BSCCO) flakes encapsulated in hBN, thus achieving desired patterns without damaging the superconducting material. Using this fabrication method, thin BSCCO flakes were decorated with complementary superconducting wires that reproducibly demonstrated single-photon sensitivity at telecommunication wavelengths (1550 nm) at operating temperatures of 20–25 K.

These detectors achieved a detection efficiency (DE) governed by the in-coupling efficiency, absorption and voltage generation probability, on the order of DE~10^−4^. Their dynamical behavior is characterized by nanosecond-scale recovery times, while their dark-count rate (a reference electronic response without detectable optical signal) is approximately 10^3^ counts per second, proving their potential for practical applications in single-photon detection [12,13]. This new generation of SNSPDs can operate with simpler cooling systems; however, the periodic pattern size is currently limited to a few tens of micrometers due to the degradation of BSCCO during the fabrication process [12,13].

The next challenge is enhancing the absorption efficiency of the nanowire pattern. One of the previously demonstrated approaches is embedding the superconducting nanowires into an optical cavity [14,15]. Our previous studies have shown that in addition to continuous optical nanocavities, significant improvements can be achieved by integrating periodic nanoplasmonic patterns into NbN-based SNSPDs [16,17,18,19]. Integration of plasmonic patterns enables the application of relatively high (wavelength-scaled) periodic nanowire patterns in a wide wavelength range and within a specific range of incidence angles, while maintaining high efficiency (>90%) [20,21,22]. The large period allows for reduced kinetic inductance, if the pattern size remains fixed, as the total nanowire length decreases. This effect is particularly relevant for NbN-based patterns, where the high kinetic inductance has a significant impact on the detector speed [23], though it is advantageously not crucial for BSCCO patterns of two-orders-of-magnitude lower kinetic inductance.

By combining previously demonstrated methods, nanocavities could be fabricated using conventional lithography, while the hBN-protected BSCCO pattern, which could be processed with He ion lithography, could be transferred by ensuring synchronization with the nanocavity array [12,13]. It should be emphasized that, although the complete hBN-protected BSCCO/plasmonic nanocavity array integrated SNSPD proposed here has not yet been experimentally realized, the individual feature sizes considered in the optimization are not incompatible with existing BSCCO nanostructuring approaches [12,13]. The main fabrication challenge is the realization of a periodically aligned hybrid architecture combining the BSCCO/hBN detector pattern with the Au nanocavity array. Accordingly, the present structures should be regarded as numerically optimized target geometries for future experimental implementation.

In this study, the existing numerical optimization strategy [20,21,22] and analysis methods are adapted for an anisotropic high-critical-temperature cuprate superconducting platform. In contrast to NbN, BSCCO was modeled using its full anisotropic permittivity tensor, together with the hBN encapsulation and the residual insulating BSCCO regions imposed by the experimentally demonstrated fabrication method. This anisotropic and fabrication-constrained material system modifies the useful absorption in the superconducting stripes, the competitive absorption in the plasmonic Au components, the resonance conditions and hence the resulting optimal geometrical parameter ranges.

Accordingly, the numerical optimization of BSCCO-based SNSPDs integrated with plasmonic patterns was performed to maximize the absorptance in the nanowires exclusively, thereby determining and optimizing an important optical contribution to the detector overall to make its operation more efficient at telecommunication wavelengths in the C-band.

The higher possible operational temperature and predicted high absorptance make these SNSPDs promising for integrated photonic circuits; moreover, as on-chip optical components they may pave the way for novel quantum optical applications.

## 2. Materials and Methods

The SNSPD geometries were designed numerically using the RF module of FEM-based COMSOL Multiphysics 6.0 software package (COMSOL AB, Stockholm, Sweden), by solving the full-wave Maxwell equations, considering the material’s dispersion by importing tabulated datasets [24,25]. The optical response of different device compositions, specifically the absorptance (arising as a loss from resistive heating in materials with non-zero imaginary parts of their permittivity), reflectance, and transmittance (computed as the outgoing power density integrated on port boundaries in the forward and backward directions), was determined as a function of wavelength and angle of incidence by using single unit cells of the inspected periodic patterns, terminated with vertical Floquet periodic boundary conditions.

The superconducting material segment was a 15 nm thick BSCCO layer (calculations for 30 nm thick wires are presented in the Supplementary Materials). Superconducting BSCCO wires were separated by interleaving residual BSCCO, arising after etching and acting as an insulator (reBSCCO). In the model, reBSCCO was treated as an insulating region by setting the imaginary part of its permittivity close to zero. The complete BSCCO layer was covered by a 20 nm hBN film, and both were defined by their full permittivity tensors accounting for the thin film anisotropy [26,27] (Figure S1 in Supplementary Materials). To evaluate the robustness of the numerical results against uncertainties in the optical material parameters, we performed an additional sensitivity analysis by varying the assumed material properties of BSCCO, hBN, and reBSCCO. The corresponding results are presented in the Supplementary Material (Figures S9 and S10).

The optimization of the geometry was realized to maximize the amount of power absorbed in the BSCCO wires (BSCCO absorptance) at 1550 nm, which is an important wavelength for telecommunication applications. Normal incidence was assumed during the optimization performed by using p-polarized light in an S-configuration [20].

**Figure 1:**
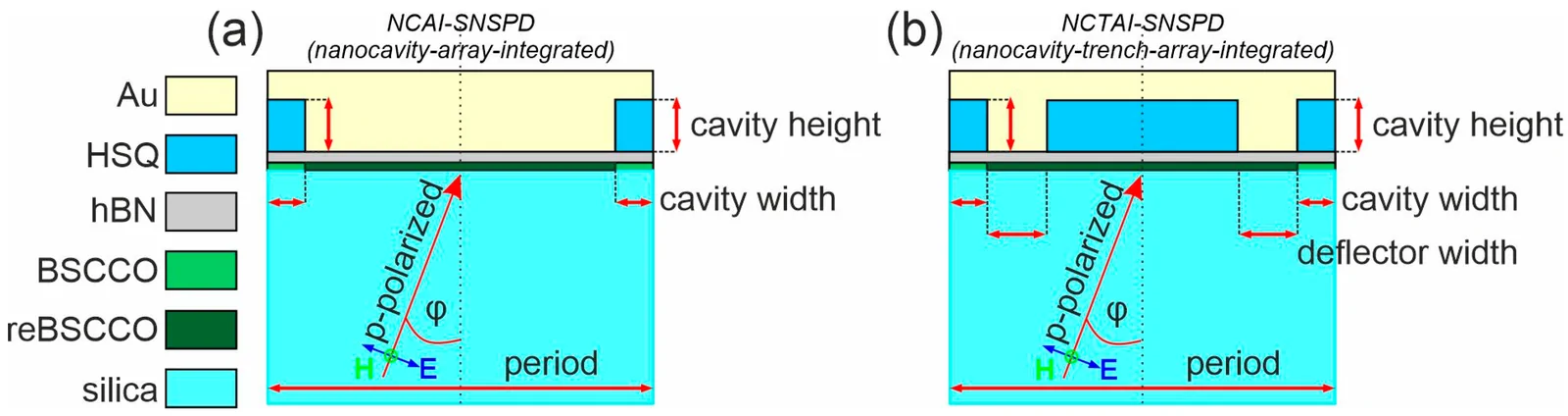
Schematic of the unit cells of the inspected NCAI-SNSPD (**a**) and NCTAI-SNSPD (**b**) periodic structures, indicating the illumination by p-polarized light and the parameters varied during optimization.

In this study, two types of SNSPD device compositions were investigated (Figure 1). In both compositions the 15 nm thick meandered BSCCO wire is positioned onto a silica substrate and covered by the 20 nm hBN and a Hydrogen silsesquioxane (HSQ) layer, with a thickness varied in the [10 nm, 800 nm] interval during optimization. On top of the HSQ layer a fixed 60 nm thick gold reflector closes an optical nanocavity that is filled with HSQ. In the first inspected device composition the superconducting wires were separated by bulk gold segments laterally, forming a nanocavity array (NCAI—nanocavity array integrated SNSPD), with a wire-to-wire distance that was varied in the [800 nm, 1200 nm] interval. In the second device composition the width of the lateral walls of the cavities was also varied symmetrically, thereby allowing for “opening an empty trench array”. The HSQ-filled cavities and trenches create a nanocavity trench array (NCTAI—nanocavity trench array integrated) SNSPD. Both inspected composition types are based on integrated devices efficiently optimized in our previous studies to improve NbN-based SNSPD absorptance, when it was shown that by introducing a secondary nanocavity (trench) array, even greater absorptance can be achieved, due to the minimized competitive gold absorptance [20,21,22].

The geometrical parameters of both compositions were optimized using the COMSOL Multiphysics Optimization Module integrated with an in-house-developed GLOBAL algorithm [28].

During optimization as a first step, a Monte Carlo simulation was conducted to determine the period, nanocavity height, (deflector width) and wire width in NCAI (NCTAI) SNSPD, that could maximize the achievable absorptance. As a second step a local search was performed to find the global maximum in the inspected geometrical parameter region (for further details please see Supplementary Materials, including the mesh convergence study of Figure S12). The objective function was defined to maximize optical power absorbed in the superconducting BSCCO regions. Accordingly, the optimization target in this study is the BSCCO absorptance, rather than the detector efficiency. The simulations do not include optical coupling losses associated with a specific experimental in-coupling configuration, nor do they model internal detection probability, hotspot formation, current redistribution, timing jitter, dark-count generation, or recovery dynamics. The far-field and near-field responses of the optimized device, as well as the optical response including the simulated absorptance, as the predominant component of the detection efficiency across the inspected spectral range, were thoroughly investigated for the BSCCO devices to uncover the underlying physics. The absorptance of optimized devices was compared to those of the standalone BSCCO meander in the simple optical cavity (Figures S2 and S3 and Table S1 in the Supplementary Materials).

Thereafter the optimized devices were inspected in the 1000–2000 nm band, applying tilting as well, to map their dispersion characteristics. The dispersion characteristics of the two integrated device compositions taken in the p-to-S configuration were compared to each other in order to characterize their potential as efficient and fast single-photon counting devices at 1550 nm.

## 3. Results and Discussion

The present numerical study optimizes only the electromagnetic absorption in the superconducting BSCCO regions. In an SNSPD, the system detection efficiency is determined by the combined effect of optical in-coupling into the active area, absorptance in the superconducting nanowire, internal detection efficiency qualifying the probability of electric signal generations, which depends on hotspot formation efficiency, and current-density distribution. In addition, the electrical performance of the device has a strong impact on the readout conditions, including the timing jitter, dark-count rate, and recovery dynamics. Consequently, the absorptance values reported here should be interpreted as optical figures of merit of the proposed plasmonic BSCCO-based geometries.

### 3.1. Reference Configurations

To define a reference for our optimized nanostructure-integrated SNSPDs comparable to the optimized geometries, compositions involving (i) a periodic, 100 nm wide BSCCO meander on a silica substrate (ii) covered with an optical cavity were first investigated (Figures S2 and S3 in the Supplementary Materials).

In the case of a periodic BSCCO meander, low BSCCO absorptance of 0.014 is achieved at 1550 nm at perpendicular incidence, which is accompanied by a high transmittance of 0.956. The absorptance can be slightly increased by increasing the operation wavelength, due to the increasing imaginary part of BSCCO permittivity. Oblique incidence is also advantageous in this configuration, as it is demonstrated by reaching 0.019 absorptance at an angle of incidence of 59°, which is promoted by the mode propagating on the BSCCO slab (Figure S2).

The optical cavity (OC) is formed by placing a 60 nm gold reflector above the BSCCO meander (Figure S3). To maximize absorptance, the cavity length is tuned to 225 nm. At 1550 nm and at perpendicular incidence, 0.041 absorptance is achieved, which is accompanied by nearly zero transmittance, a minimal 0.027 competitive Au absorptance, and a high, 0.921 reflectance. The absorptance can be slightly increased by increasing the operation wavelength; however, tilting is not advantageous in this composition (Figure S3). 

### 3.2. Nanocavity Array

Dividing the dielectric cavity with vertical gold segments creates a nanocavity array, which is a common element of the proposed plasmonic structure integrated SNSPDs (Figure 1).

According to the initial Monte Carlo simulations in both the nanocavity-array integrated (NCAI-SNSPD) and the nanocavity-trench-array integrated (NCTAI-SNSPD) compositions, two absorptance peaks emerge as a function of nanocavity length, corresponding to the λ/(4 × n_eff_) and 3 × λ/(4 × n_eff_) modes of the nanocavities (Figures S4a and S5a).

In the case of NCAI-SNSPD, absorptance plateaus were observed for larger cavity widths and smaller periods, i.e., corresponding to increasing superconducting pattern filling factor (Figure S4b,c). The result of plateauing indicates that a significant reduction in period or increase in wire width is not accompanied by a significant increase in absorptance. A trade-off exists for this composition; therefore, using an intermediate period, allowing for a reduction in charge-crowding effect, with only a slightly compromised decrease in absorptance, is reasonable to consider. In the case of NCTAI-SNSPD, a global maximum emerges at small/large/intermediate cavity width/period/deflector width, respectively (Figure S5 in the Supplementary Materials).

**Figure 2:**
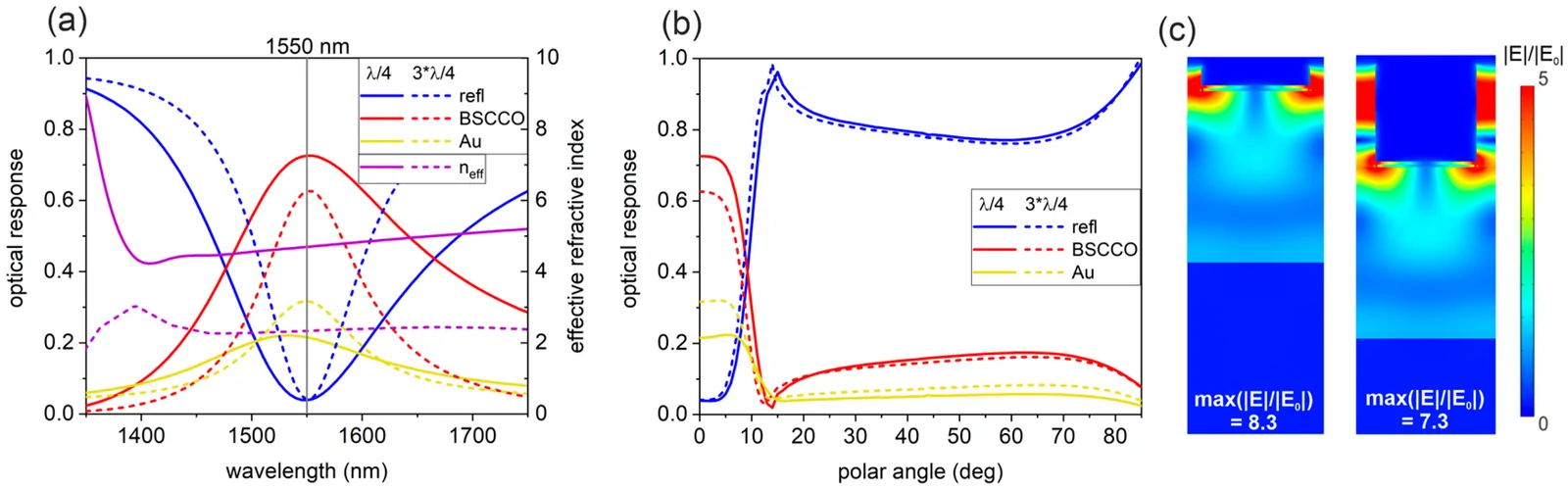
Optical response of optimized NCAI-SNSPD composition in p-to-S configuration. (**a**) Optical response and effective refractive index spectrum and (**b**) polar angle dependence. Solid: ~λ/(4 × n_eff_) mode dashed: ~3 × λ/(4 × n_eff_) mode. (**c**) Time-averaged near-field enhancement of NCAI-SNSPD optimized to resonate in the (left) λ/(4 × n_eff_) mode and (right) 3 × λ/(4 × n_eff_) mode. Please note that the color scale was saturated at a value of 5 to enhance the visibility of fine structural details; the actual maximum near-field intensity exceeds this value.

**Figure 3:**
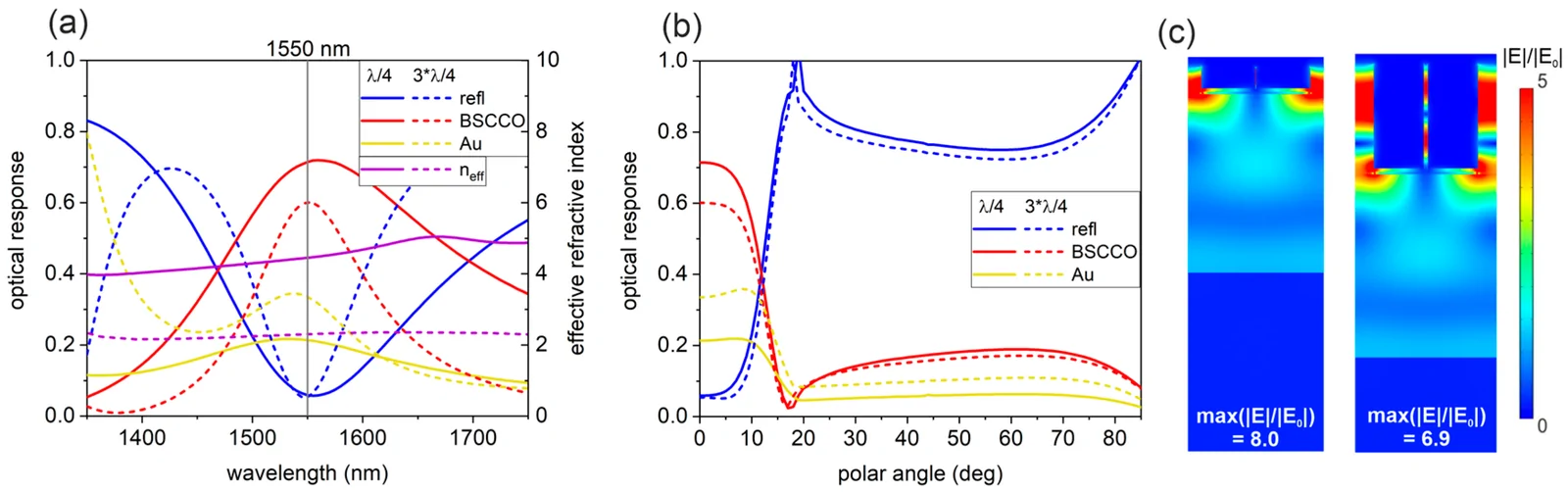
Optical response of optimized NCTAI-SNSPD composition in p-to-S configuration. (**a**) Optical response and effective refractive index spectrum and (**b**) polar angle dependence. Solid: ~λ/(4 × n_eff_) mode dashed: ~3 × λ/(4 × n_eff_) mode. (**c**) Time-averaged near-field enhancement of NCTAI-SNSPD optimized to resonate in the (left) λ/(4 × n_eff_) mode and (right) 3 × λ/(4 × n_eff_) mode. Please note that the color scale was saturated at a value of 5 to enhance the visibility of fine structural details; the actual maximum near-field intensity exceeds this value.

Following Monte Carlo simulations, local searches were performed with NCAI-SNSPD and NCTAI-SNSPD around the two absorptance peaks corresponding to different nanocavity resonant modes (Figure 2 and Figure 3). The NCAI-SNSPD optimized to resonate in the λ/(4 × n_eff_) cavity mode exhibits a broad Lorentzian resonance in absorptance centered around 1550 nm (Figure 2a solid lines). The peak absorptance of NCAI-SNSPD is 0.726, which is accompanied by a low reflectance of 0.039 and negligible transmittance, and 0.216 competitive gold absorptance. The BSCCO absorptance reaches a global maximum at perpendicular incidence, as indicated by the polar angle-dependent optical response at the center of a pass band (Figure 2b solid lines). In the close vicinity of the BSCCO nanowire, 8.3-fold maximum near-field enhancement is reached (Figure 2c, left).

When optimized for the cavity mode resonating in a nanocavity of ~3 × λ/(4 × n_eff_) length, the NCAI-SNSPD shows a smaller and narrower absorptance peak centered around 1550 nm (Figure 2a, dashed lines). The higher Q-factor is typical for higher-order plasmonic resonances. The maximal absorptance value is 0.626, accompanied by a small, but non-negligible, reflectance (0.041), and a higher gold absorptance (0.317), when compared to those achieved in NCAI-SNSPD optimized for resonance in the λ/(4 × n_eff_) cavity mode. The polar angle dependence of the optical responses in the case of the higher-order mode closely resembles that observed in the case of the λ/(4 × n_eff_) cavity resonant mode, with consistently smaller absorptance values and extrema occurring at slightly smaller polar angles (Figure 2b dashed lines). The highest absorptance is achieved at perpendicular incidence, and similarly at the center of a pass band, which is slightly narrower in accordance with the higher quality factor. A 7.3-fold maximum near-field enhancement is achievable, which is slightly smaller compared to that reached in NCAI-SNSPD optimized to resonate in the λ/(4 × n_eff_) cavity mode (Figure 2c).

Standard retrieval calculations [29] indicate that the optimal cavity lengths correspond to ~ λ/(4 × n_eff_) and 3 × λ/(4 × n_eff_) with good precision (Figure 2a, purple lines).

The NCTAI-SNSPD is optimized to resonate in the λ/(4 × n_eff_) and 3 × λ/(4 × n_eff_) modes as well (Figure 3). In the case of the lower order mode the absorptance spectrum of the optimized NCTAI-SNSPD reveals a similar pass band centered at 1550 nm as it was shown in the optimized NCAI-SNSPDs (Figure 3a, solid lines).

The global maximum in the absorptance spectrum is 0.715, which is accompanied by a small reflectance (0.059), negligible transmittance, and a relatively large competitive gold absorptance (0.214). This maximum is attained at perpendicular incidence (Figure 3b solid lines). At larger polar angles, the absorptance rapidly decreases, while at intermediate values, it exhibits a wider, shallower local absorptance maximum. At the global maximum an 8-fold near-field enhancement is observable (Figure 3c).

When optimized to resonate in the 3 × λ/(4 × n_eff_) mode, the NCTAI-SNSPD exhibits a significantly narrower peak in the absorptance spectrum, which is precisely centered at 1550 nm (Figure 3a, dashed lines). The maximal absorptance value is 0.601, accompanied by a small (0.054) reflectance and a more significant competitive gold absorptance (0.335).

This maximum is reached at perpendicular incidence, with the absorptance rapidly decreasing at small tilting, while a small local maximum appears at large polar angles (Figure 3b, dashed lines). The near-field enhancement at the global maximum is 6.9-fold, which is slightly smaller compared to that reached in NCTAI-SNSPD optimized to resonate in the λ/(4 × n_eff_) cavity mode (Figure 3c).

Standard retrieval performed for perpendicular incidence reveals that the optimized nanocavity structure has an effective refractive index, corresponding to ~λ/(4 × n_eff_) and ~3 × λ/(4 × n_eff_) at the first and third nanocavity resonance (Figure 3a, purple lines).

### 3.3. Comparative Study

The absorptance spectra and polar angle dependence characteristics of NCAI-SNSPD and NCTAI-SNSPD reveal similar resonant modes on the optimized integrated plasmonic gratings.

**Figure 4:**
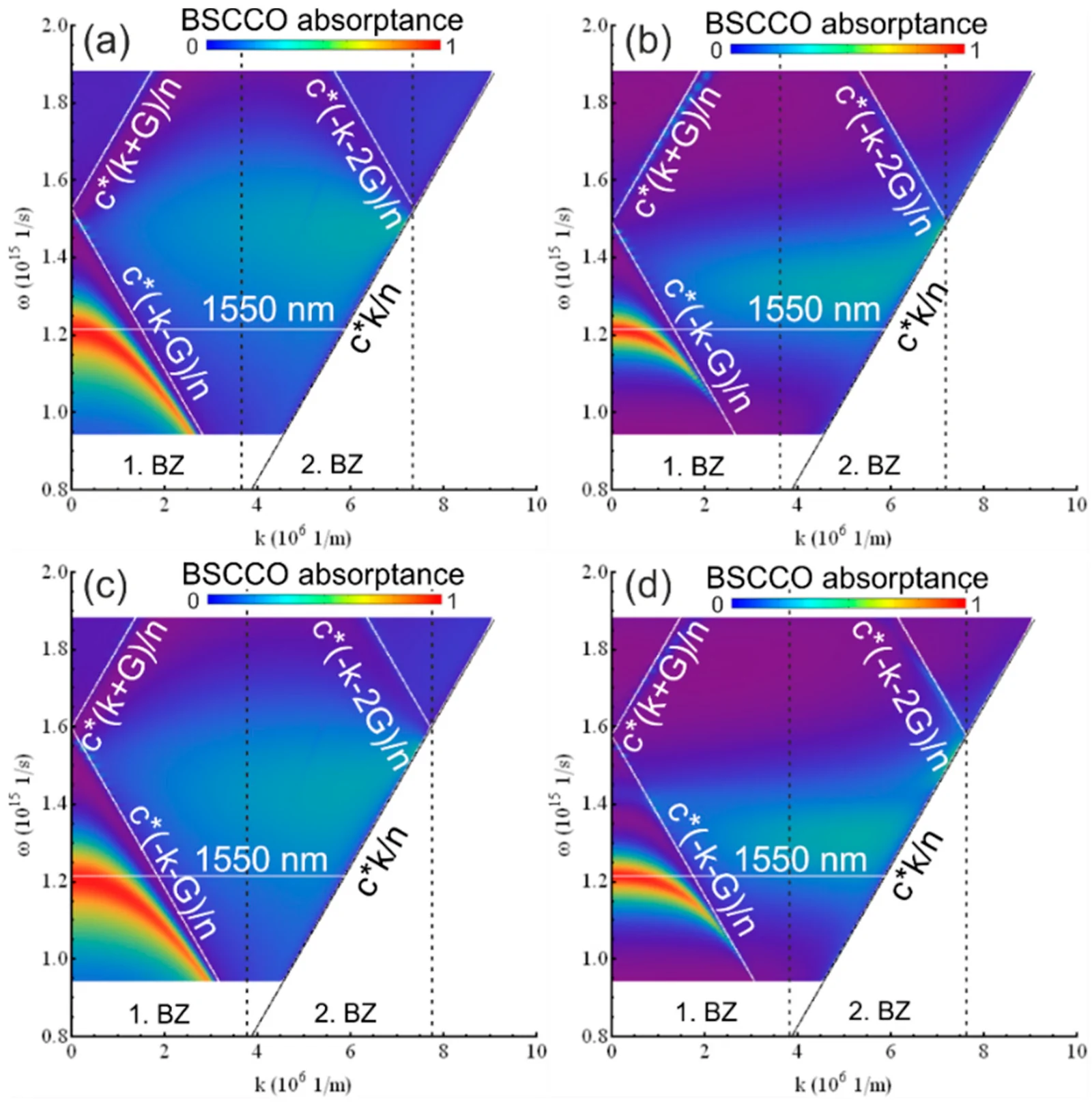
Dispersion diagram of BSCCO absorptance of (**a**) NCAI-SNSPD in λ/(4 × n_eff_) mode, (**b**) NCAI-SNSPD in 3 × λ/(4 × n_eff_) mode, (**c**) NCTAI-SNSPD in λ/(4×n_eff_) mode, and (**d**) NCTAI-SNSPD in 3 × λ/(4 × n_eff_) mode.

To analyze the underlying physics, the dispersion diagrams of the optimized compositions in the p-to-S configuration are compared (Figure 4). The optimal NCAI-SNSPD composition exhibits a well-defined low-energy plasmonic mode below the first-order grating coupling (Figure 4a,b). The branches are folded back into the first Brillouin zone (BZ) and result in a single Lorentzian resonance in the wavelength dependence at perpendicular incidence (Figure 2a). The absorptance remains large over a relatively wide polar angle interval (at incidence angles of 10° and 9°, the absorptance decreases to 1/e of its maximum value for the NCAI-SNSPD λ/(4 × n_eff_), and 3 × λ/(4 × n_eff_) respectively) but sharply decreases below 0.010 around 13° tilting, which is consistent with the edge of this pass band being tuned to 1550 nm. Along the grating-coupled branches the absorptance of BSCCO is diminished; however, at large tilting the absorptance is re-enhanced in a wide polar angle and wavelength interval across the second Brillouin zone. This corresponds to the plasmonic Brewster angle (PBA), whose condition is met when the grating is impedance-matched with the substrate and results in anomalous energy squeezing into and tunneling through the nanocavity array, thereby suppressing reflectance and enhancing absorptance [30,31]. The maximum of the absorptance in PBA region is centered below 1550 nm. The PBA is calculated as (Tables S4 and S5 in the Supplementary Materials) [30,31]

PBA = acos(w_cavity_ ∙ λ/(n ∙ p ∙ λ_plasmon_)), (1)

where w_cavity_ is the width of cavity, λ/n_silica_ is the wavelength incident in silica substrate, p is the period of the unit cell and λ_plasmon_ is the wavelength of propagating surface plasmon polariton (SPP), which is approximated with the value calculated at the interface of semi-infinite media. It can be noted that both the propagating plasmon’s resonance and the PBA-related peaks are significantly narrower in frequency in the optimized composition with 3 × λ/(4 × n_eff_) cavity length, which indicates high-Q modes of longer lifetime. This is also confirmed by eigenmode calculations, which results in ω_NCAI_1×λ/4_ = (1.240 + 0.090i) × 10^15^ 1/s and ω_NCAI_3×λ/4_ = (1.220 + 0.050i) × 10^15^ 1/s (1522 nm and 1548 nm, respectively) [32].

The optimal NCTAI-SNSPD compositions exhibit dispersion diagrams similar to those of their respective NCAI-SNSPD counterparts. A plasmonic mode below the first-order grating coupling results in a pass band around 1550 nm and perpendicular incidence. In the second BZ a PBA phenomenon of distinctly different dominance appears. In the case of 3 × λ/(4 × n_eff_) nanocavity length the resonance bandwidth is significantly smaller. The propagating plasmonic mode band is folded back into the first Brillouin zone, at similar frequency as in the case of NCAI-SNSPD (ω_NCTAI_1×λ/4_ = (1.230 + 0.110i) × 10^15^ 1/s and ω_NCTAI_3×λ/4_ = (1.220 + 0.060i) × 10^15^ 1/s), while the first-order grating-coupled SPP branch is observable in NCTAI-SNSPD as well.

Comparing the optimized integrated devices, the NCAI-SNSPD exhibits slightly larger BSCCO absorptance along with smaller reflectance than the NCTAI-SNSPD. The exact absorptance difference is 0.011 and 0.025 for the device operating in λ/(4 × n_eff_) mode and 3 × λ/(4 × n_eff_) mode, respectively. While the NCAI-SNSPD exhibits slightly higher BSCCO absorptance, it is achieved with a larger filling factor compared to the NCTAI-SNSPD. Although it was not directly verified in the presented optical simulations, in NCTAI-SNSPD this reduced filling factor is potentially beneficial for the electrical performance of the detector by mitigating charge-crowding effects and allowing a larger critical current inferred from geometrical considerations and SNSPD development trends [33].

In general, it can be concluded that the NCAI-SNSPD and NCTAI-SNSPD compositions exhibit very similar optical responses. This is because the geometry of the optimal systems is also very similar, since in optimized NCTAI-SNSPD configurations, the deflectors are so wide that only a narrow trench is created as a secondary periodic element, i.e., the optimal NCTAI-SNSPD geometry approaches the NCAI-SNSPD geometry. The appearance of the secondary nanocavity inherently causes a difference in the optical response; therefore, it remains the subject of ongoing studies, whether a slightly smaller filling factor is optimal in NCTAI-SNSPD compositions.

The absorptance of BSCCO stripes can be further enhanced by increasing their thickness to 30 nm (while decreasing the encapsulating hBN thickness to 5 nm at the same time to keep the complete thickness constant) and re-optimizing the geometry. After that the main characteristics remain the same for all inspected compositions and configurations, namely a single plasmonic mode centered around 1550 nm appears and creates a pass band (Figures S6–S8 in the Supplementary Materials). With 30 nm BSCCO thickness the reflectance decreases below 0.010 while the absorptance reaches 0.867 and 0.798 in the NCAI-SNSPD composition operating with the λ/(4 × n_eff_) and 3 × λ/(4 × n_eff_) modes, respectively, while 0.844 and 0.783 absorptance is attainable with NCTAI-SNSPD composition in λ/(4 × n_eff_) and 3 × λ/(4 × n_eff_) resonant mode configurations, respectively.

## 4. Conclusions

In conclusion, the plasmonic grating integrated SNSPD based on a BSCCO nanowire pattern was numerically optimized to maximize absorptance at 1550 nm and perpendicular incidence. The nanocavity array integrated SNSPD optical responses demonstrate the capability to increase absorptance by more than an order of magnitude compared to a simple optimized optical nanocavity and an embedded meandered superconducting BSCCO pattern with a similar 0.1 filling factor. Both the λ/(4 × n_eff_) and 3 × λ/(4 × n_eff_)-scaled nanocavity arrays are capable of maximizing absorptance, but the lower order cavity resonance in a shorter nanocavity consistently results in larger BSCCO absorptance with smaller competitive gold absorptance and spurious reflectance.

While the NCAI-SNSPD possesses larger BSCCO absorptance, the NCTAI-SNSPD remains an optically competitive design with a smaller filling factor. This smaller filling factor may be beneficial for superconducting charge transport and critical-current performance, but leveraging these advantages requires further experimental investigations on electrothermal phenomena that may have a strong impact on the current density involved in the detection phenomenon.

## Data Availability

The original contributions presented in this study are included in the article/Supplementary Material. Further inquiries can be directed to the corresponding author.

## Supplementary Materials

The following supporting information can be downloaded at https://www.mdpi.com/article/10.3390/mi17080887/s1, Figure S1: Wavelength-dependent dielectric permittivity of BSCCO and hBN; Figure S2: Optical response of standalone wire; Figure S3: Optical response of optical cavity and gold reflector; Table S1: Optical response of the inspected reference detector compositions; Table S2: Name, interval and description of optimized parameters of NCAI-SNSPDs; Table S3: Name, interval and description of optimized parameters of NCTAI-SNSPDs; Figure S4: Monte Carlo simulation of NCAI-SNSPD; Figure S5: Monte Carlo simulation of NCTAI-SNSPD; Table S4: Optical response of optimized SNSPD compositions at 1550 nm with 15 nm thick wire; Figure S6: Optical response of optimized NCAI-SNSPD composition in p-to-S configuration; Figure S7: Optical response of optimized NCTAI-SNSPD composition in p-to-S configuration; Figure S8: Dispersion diagram of BSCCO absorptance; Table S5: Optical response of optimized SNSPD compositions with 30 nm thick wire; Figure S9. BSCCO absorptance with varied hBN layer thickness. BSCCO thickness = 15 nm; Figure S10. Histogram of BSCCO absorptance values calculated for the optimized geometry with material permittivities varied by ±20%; Figure S11. Histogram of BSCCO absorptance values calculated for the optimized geometry with geometric parameters varied by ±5-10-20%; Figure S12. Convergence study in the case of NCAI-SNSPD with λ/(4 × neff) cavity length.

## Abbreviations

The following abbreviations are used in this manuscript:

SNSPD	superconducting nanowire single-photon detectors
NbN	niobium-nitride
BSCCO	Bi_2_Sr_2_CaCu_2_O_8+δ_
DE	detection efficiency
FEM	finite element method
hBN	Hexagonal Boron Nitride
HSQ	Hydrogen silsesuioxane
NCAI	nanocavity array integrated
NCTAI	nanocavity trench array integrated
OC	optical cavity
BZ	Brillouine zone
PBA	plasmonic Brewster angle
SPP	propagating (surface) plasmon polariton
