# Supplementary material for: Numerical Design of Plasmonic Structure Integrated Superconducting Nanowire Single-Photon Detector Constructed with BSCCO Patterns
Source: Micromachines (Basel). 2026 Jul 25;17(8):887. doi: 10.3390/mi17080887 (PMC13515199; doi:10.3390/mi17080887)
Supplement: Supplementary file 1 [file micromachines-17-00887-s001.zip › micromachines-4373344-supplementary.pdf]

Supplementary Information

Numerical design of plasmonic structure integrated superconducting nanowire single-photon detector constructed with BSCCO patterns

András Szenes[1,2] , László Pothorcki[1] , Balázs Bánhelyi[2,3], Mária Csete[1,2]

[1] Department of Optics and Quantum Electronics, University of Szeged, 6720, Dóm tér 9, Szeged, Hungary
[2] HUN-REN Wigner Research Center of Physics, Konkoly-Thege Miklós út 29-33, Budapest, Hungary
[3] Department of Computational Optimization, University of Szeged, 6720, Árpád tér 2, Szeged, Hungary

Material dispersion of BSCCO and hBN

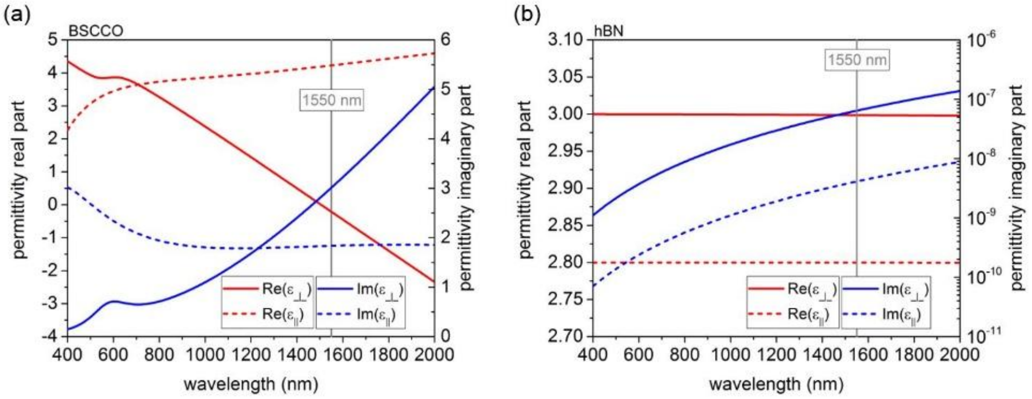

Figure S1. Wavelength-dependent dielectric permittivity of (a) BSCCO and (b) hBN used in the simulations.

For BSCCO and hBN, their anisotropic optical properties were considered using a 3x3 tensor [S1, S2]. The elements of the complex diagonal tensor normal and parallel to the layer ($\varepsilon_{\perp}$ and $\varepsilon_{\|}$, respectively) are shown in Figure S1 in the wavelength range of 400-2000 nm. The imaginary part of the hBN is very small in this range, so its absorptance is close to zero; hence, it is not plotted in the main text figures.

Optical response of reference compositions

As a reference system for the presented NCAI-SNSPD and NCTAI-SNSDP devices, two simpler device compositions without integrated plasmonic structures were modeled.

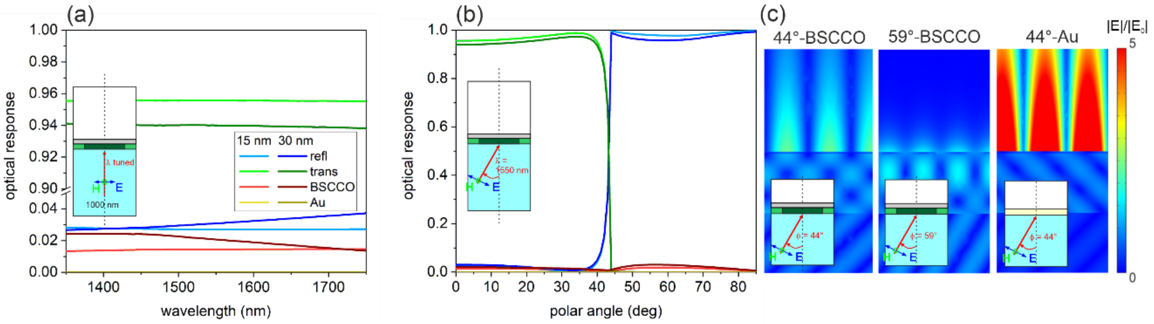

Figure S2. Optical response of stand-alone hBN-protected intact BSCCO wire and its leftover insulator (reBSCCO) complementary pattern on silica substrate (air is the bounding medium): (a) wavelength and (b) polar angle dependence. Insets: Schematic of the geometry and configuration used for illumination with p-polarized light. (c) Evanescent a plasmon-like surface modes at the maximum reflectance (φ=44°) and absorptance (φ=59°) and propagating plasmon polariton mode on a 35 nm thick gold slab for comparison.

The absorptance of a BSCCO wire of 1/10 filling factor as a function of wavelength and angle of incidence was investigated (Figure S2). For substrate-side illumination, a low absorptance (less than 0.015,) was obtained, over the investigated wavelength range, though its value increases monotonically with wavelength. The reflectance is also very low, accordingly the dominant fraction of the incident power is transmitted. By varying the angle of incidence at 1550 nm the absorptance and reflectance decrease monotonically at small tilts and above the attenuated total reflection (ATR); at 44°, the absorptance shows a global maximum (at 59°), while the transmittance rapidly decreases to zero. At 44°/59°, an evanescent mode /SPP-like propagating mode on the BSCCO layer is visible (Figure S2c). According to additional calculations, the near-field enhancement reached at the ATR of the BSCCO layer is significantly smaller compared to the near-field enhancement achievable at the SPP resonance of the gold thin film (Figure S2 insets). The commensurable near-field enhancement at the ATR and at the global absorptance maximum is the result of the BSCCO dielectric properties, differ from noble metals (Figure S1).

To enhance the absorptance, the BSCCO layer thickness was increased to 30 nm. In this case not only the absorptance values but also the optical response tendencies change. Namely, the absorptance is the largest at a perpendicular incidence (0.021, Figure S2a) and a monotonous decrease is observable. The polar angle dependence is similar with slightly increased absorptance throughout the complete inspected tilting range (Figure 2Sb).

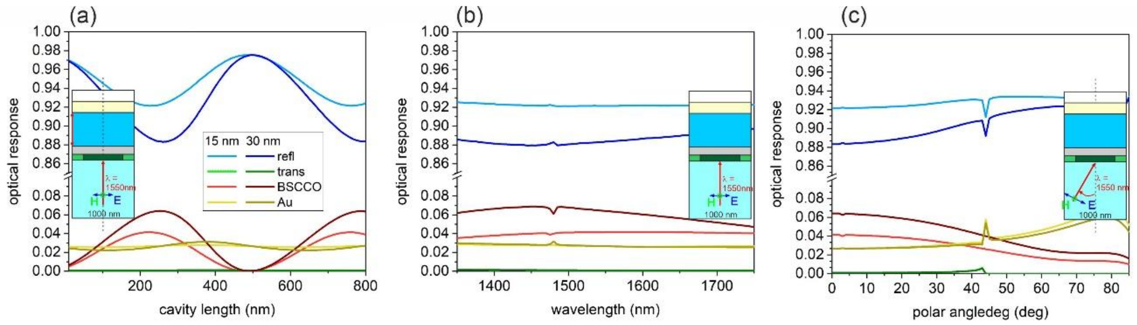

Figure S3. Optical response of an optical cavity and gold reflector (60 nm) covering a hBN-protected intact BSCCO wire pattern and its leftover insulator (reBSCCO) complementary pattern: (a) cavity length tuning, (b) wavelength and (c) polar angle dependence. Insets: Schematic of the geometry and illumination configuration used for illumination with p-polarized light in S-configuration (plane of incidence is perpendicular to the patterns).

Next, a 60 nm thick gold reflector terminated the BSCCO layer covered with an HSQ spacer forming an optical cavity (OC). First, for perpendicular incidence and a fixed wavelength of 1550 nm, the optimal thickness of the dielectric spacer was determined by maximizing the achievable absorptance (Figure S3a). It was found that thicknesses of 225 nm and 760 nm both provide a maximal absorptance value of approximately 0.041, with high reminiscent reflectance, low gold absorptance and negligible transmittance. With a fixed 225 nm cavity length, the BSCCO absorptance monotonically increased with increasing wavelength due to the slightly/significantly increasing imaginary part of the parallel/normal component of the permittivity (Figures S1a and S3b), with decreasing complementary reflectance in the inspected wavelength interval. At 1550 nm wavelength, the impact of tilting was examined and it was shown that a monotonic decrease in absorptance is accompanied by a slightly and considerably increasing reflectance and gold absorptance, due to the gradually modifying reflection coefficient of the multilayer for the inspected p-polarized light.

By increasing the BSCCO thickness to 30 nm, the optimal height of the OC is increased to 255 and 785 nm corresponding the one and three-quarter wavelength cavity. At perpendicular incidence, the absorptance is 0.064 and a global maximum is observable by increasing the wavelength, with a smaller reflectance and similar gold absorptance compared to the case of 15 nm BSCCO thickness. At 1550 nm, by changing the tilting, the BSCCO absorptance gradually decreases, whereas the gold absorptance increases, since the reflection coefficient of the multilayer for the inspected p-polarization was modified. The absorptance is enhanced and the reflectance is suppressed compared to the 15 nm counterpart BSCCO film.

	meander (15 nm)			meander (30 nm)			OC + meander (15 nm)	OC + meander (30 nm)
	1550 nm, 0°	1550 nm, 44°	1550 nm, 59°	1550 nm, 0°	1550 nm, 43°	1550 nm, 56°	1550 nm, 0°	1550 nm, 0°
BSCCO absorptance	1.4	0.5	1.9	2.1	0.8	3.1	4.1	6.4
Au absorptance	-	-	-	-	-	-	2.7	2.7
Reflectance	2.7	99.5	98.1	3.1	99.2	95.9	92.1	88.3
Transmittance	95.6	0	0	94.0	0	0	0.1	0.1

Table S1. Optical response of the inspected reference detector compositions. Geometrical parameters of the optical cavity + BSCCO layer are: 1000 nm BSCCO meander period and 225 nm and 255 nm optical cavity length for 15 nm and 30 nm BSCCO thickness, respectively.

Monte Carlo simulations of SNSPDs

As a first step of the optimization, Monte Carlo simulations were performed, i.e., the optical response of the device composition was calculated with random geometrical parameter values from a given range. In order to conclude which geometrical parameter ranges could prove to be optimal, the absorptance read-out in intact BSCCO wire was plotted as a function of specific parameters.

The parameter bounds were as follows:

NAME	INTERVAL (nm)	DESCRIPTION
h_{HSQ}	10 – 800	height of dielectric cavity, excluding the 15+20 nm / 30 + 5 nm thick BSCCO + hBN bilayer
w_{cavity}	10 – 250	width of nanocavity and intact superconducting BSCCO wire
period	800 – 1200	period of unit cell i.e. the intact BSCCO meandered pattern

Table S2. Name, interval and description of optimized parameters in case of NCAI-SNSPDs.

NAME	INTERVAL (nm)	DESCRIPTION
h_{HSQ}	10 – 800	height of dielectric cavity, excluding the 15+20 nm / 30 + 5 nm thick BSCCO + hBN bilayer
w_{cavity}	10 – 250	width of cavity and intact superconducting BSCCO wire
period	1000 – 1200	period of unit cell, i.e., the intact BSCCO meandered pattern
w_{def}	10 - (period-w_{cavity})/2	width of cavity walls (deflectors)

Table S3. Name, interval and description of optimized parameters in case of NCTAI-SNSPDs.

Initially the h_{HSQ} height interval was set to 10-800 nm, which corresponds to a quarter-wavelength-to-three-quarter wavelength nanocavity interval, i.e., 1550 nm / (4 × n_{silica}) to 3 × 1550 nm / (4 × n_{silica}). This is an approximation, since the effective optical parameters are modified in the presence of the absorbing layers. The period interval is centered around the wavelength of photonic and plasmonic modes that propagate in the medium, namely $\lambda_{photonic}$=1550 nm / n_{silica} = 1068 nm and $\lambda_{plasmonic}$= 1055 nm, supposing semi-infinite bounding gold–silica layers. The nanocavity width covered a wide interval, from 10 to 250 nm.

These intervals were extended compared to the bounds in our previous works, considering the Monte Carlo results and effective wavelength calculations, which was necessary to allow reaching the global absorptance maximum.

Indeed, based on the nanocavity-dependent absorptance, two modes can be excited in the NCAI-SNSPD compositions over the nanocavity length range under study (Figure S4). From the effective parameter retrieval analysis, it was found that these modes can be related to the modes of $\lambda/(4 \times n_{eff})$- and $3 \times \lambda/(4 \times n_{eff})$-long cavities. The $\lambda/(4 \times n_{eff})$ mode provides a higher global maximum in the BSCCO absorptance. As the nanocavity width (and also the BSCCO fill factor) increases, the absorptance increases, but changes only slightly above 110 nm width. As the period increases (and the BSCCO fill factor decreases), the absorptance decreases slowly first and then decreases rapidly above 1000 nm. Increasing the wavelength further above 1050 nm, local extrema appear, corresponding to the photonic mode.

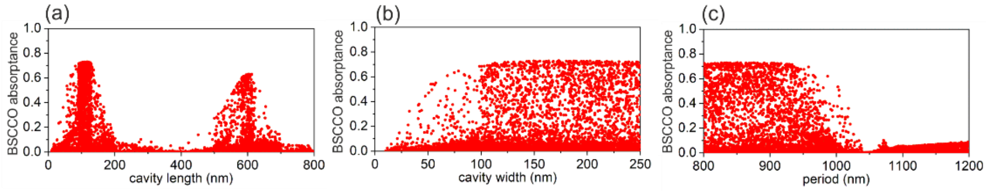

Figure S4. Monte Carlo simulation of NCAI-SNSPD. Absorptance as a function of (a) nanocavity height, (b) nanocavity width and (c) pattern period.

For the NCTAI-SNSPD compositions (Figure S5), less-well-defined maxima appear as a function of nanocavity length due to the fact that a secondary nanocavity pattern is integrated onto the detector surface. However a similarity in the two device compositions is that the $\lambda/(4 \times n_{eff})$ nanocavity modes provide the highest BSCCO absorptance. Interestingly, the period dependence of NCTAI-SNSPD is reversed compared to NCAI-SNSPD, since the absorptance takes on the global maximum at small periods. The nanocavity width shows similar dependence as in the NCAI-SNSPD composition. For the NCTAI-SNSPD compositions, the width of the nanocavity walls (deflectors) is also varying, which results in a global absorptance maximum around 400 nm, i.e., a relatively large amount of gold is optimal, which contradicts the intuitive expectations. This may be related to the coupled nanocavity and propagating modes that outperform competing trench modes, which could result in BSCCO absorptance suppression.

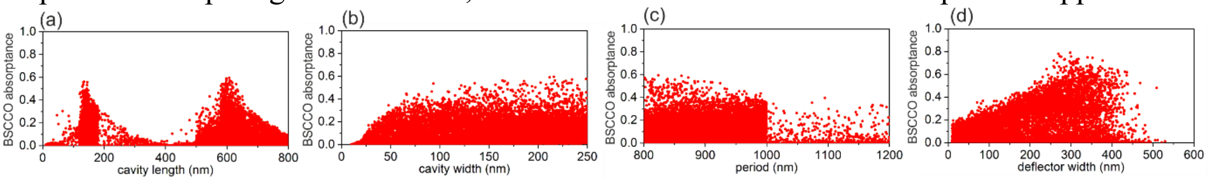

Figure S5. Monte Carlo simulation of NCTAI-SNSPD. Absorptance as a function of (a) nanocavity height, (b) nanocavity width, (c) pattern period and (d) deflector width.

For both the NCAI-SNSPD and NCTAI-SNSPD configurations, 10.000 randomly generated geometries were evaluated. The BSCCO absorptance at 1550 nm and normal incidence was calculated for each sampled geometry. This mapping revealed well-defined regions of high absorptance in the parameter space (Figure S4 and S5). These regions correspond to nanocavity lengths close to the $\lambda/(4 \times n_{eff})$ and $3 \times \lambda/(4 \times n_{eff})$ resonance conditions. For each resonance regime, the geometry yielding the largest BSCCO absorptance in the Monte Carlo scan was selected as the initial parameter set for the subsequent local optimization.

During the local optimization, the free geometrical parameters were iteratively varied around the initial solution to further increase the BSCCO absorptance. The optimization was terminated when no additional parameter variation within the applied numerical tolerance resulted in a further increase in the objective function. The geometrical parameters were varied with a numerical resolution of 0.01 nm during the final refinement step, i.e., this value should be interpreted as a merit of numerical convergence.
The resulting geometries are therefore considered the best numerically optimized solutions within the investigated parameter ranges. However, the Monte Carlo exploration does not constitute a strict mathematical proof of global optimality; the dense sampling and the subsequent local refinement reduce the probability of missing a higher-absorptance solution within the inspected parameter space.

Optimized compositions

optical response (1550 nm, 0°)	NCAI		NCTAI	
	$\lambda/(4\cdot n_{eff})$	$3\times\lambda/(4\cdot n_{eff})$	$\lambda/(4\cdot n_{eff})$	$3\times\lambda/(4\cdot n_{eff})$
BSCCO absorptance	0.726	0.626	0.715	0.601
Au absorptance	0.216	0.317	0.214	0.335
reflectance	0.039	0.041	0.059	0.054
max. near-field	5.4 (8.3)	4.3 (7.3)	5.4 (8.0)	4.3 (6.9)
n_{eff} (1550 nm)	4.7	2.3	4.5	2.3
$\lambda/(4\cdot n_{eff})$ (nm)	82.4	165.6	86.4	167.7
$3\times\lambda/(4\cdot n_{eff})$ (nm)	247.3	496.8	259.1	503.0
λ/n_{eff} (nm)	329.8	662.4	345.4	670.7
h_{HSQ} (nm)	116.9	602.0	126.8	607.6
total cavity height (nm)	131.9	617.0	141.8	622.6
period (nm)	851.0	870.1	812.6	823.2
cavity width (nm)	173.2	241.4	161.2	216.0
fill factor	20.4%	27.7%	19.8%	26.2%
PBA (°)	78.1	73.7	78.4	74.6

Table S4. Optical response of optimized SNSPD compositions at 1550 nm and perpendicular incidence of 15 nm thick BSCCO wire. The calculated effective refractive index and cavity height are also presented and the matched lengths are indicated with a green background. PBA—calculated plasmonic Brewster angle.

SNSPD compositions optimized with 30 nm thick BSCCO pattern

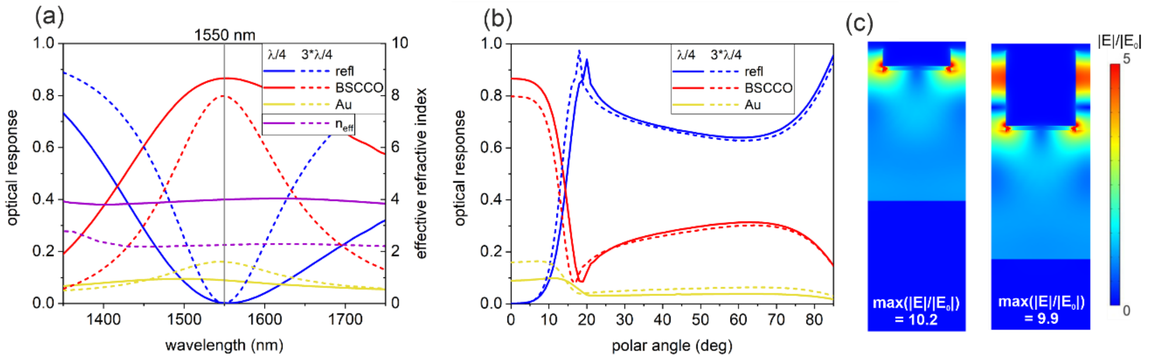

Figure S6. Optical response of optimized NCAI-SNSPD composition in p-to-S configuration. (a) Optical response and effective refractive index spectrum and (b) polar angle dependence. Solid: ~$\lambda/(4 \times n_{eff})$ mode; dashed: ~$3 \times \lambda/(4 \times n_{eff})$ mode. (c) Time-averaged near-field enhancement of NCAI-SNSPD optimized to resonate in the (left) $\lambda/(4 \times n_{eff})$ mode and (right) $3 \times \lambda/(4 \times n_{eff})$ mode. Please note that the color scale was saturated at a value of 5 to enhance the visibility of fine structural details; the actual maximum near-field intensity exceeds this value and is indicated at the bottom of the picture.

In the absorptance spectrum of NCAI-SNSPD compositions optimized with 30 nm thick BSCCO wire, a single resonance appears at the expected target wavelength of 1550 nm (Figure S6a). The optical response characteristic is similar to that of NCAI-SNSPDs optimized with a 15 nm thick BSCCO layer (Figure 2 in main text). It can be concluded that in the two configurations the one with shorter cavities provides larger and broader absorptance maximum. By changing the polar angle (at 1550 nm), after a narrow plateau, the absorptance decreases rapidly in both configurations, then reaches a smaller local maximum at large angles (Figure S6b). The near-field and effective refractive index also confirm that the nanocavity lengths are comparable to a quarter and three-quarters of the SPP modes wavelength propagating in the medium (Figure S6c).

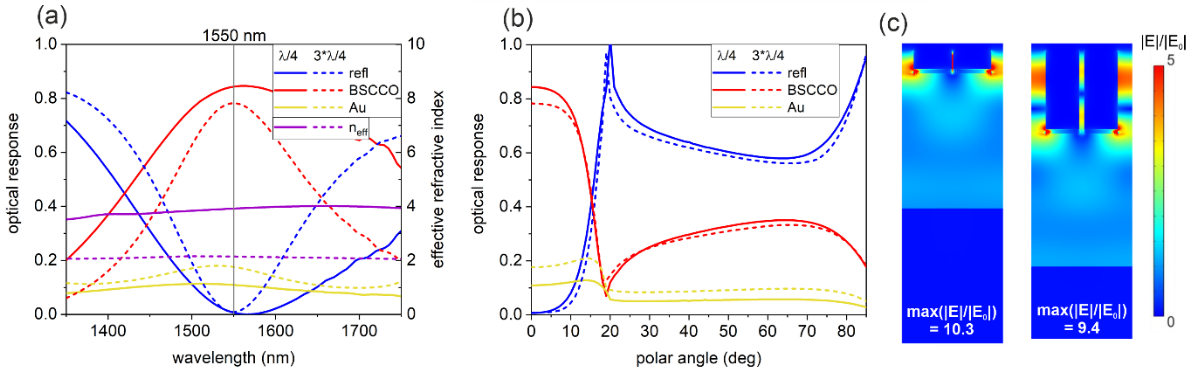

Figure S7. Optical response of optimized NCTAI-SNSPD composition in p-to-S configuration. (a) Optical response and effective refractive index spectrum and (b) polar angle dependence. Solid: ~λ/(4 × n_{eff}) mode; dashed: ~3 × λ/(4 × n_{eff}) mode. (d) Time-averaged near-field enhancement of NCTAI-SNSPD optimized to resonate in the (left) λ/(4 × n_{eff}) mode and (right) 3 × λ/(4 × n_{eff}) mode. Please note that the color scale was saturated at a value of 5 to enhance the visibility of fine structural details; the actual maximum near-field intensity exceeds this value and is indicated at the bottom of the picture.

The NCTAI-SNSPD compositions optimized with 30 nm thick wires exhibit characteristics very similar to those of NCAI-SNSPDs (Figure S7). A resonant mode-related single maximum appears in the examined wavelength interval around 1550 nm (Figure S7a), the BSCCO absorptance reaches a minimum at an incidence angle of around 20° and a local maximum at larger incidence angles (Figure S7b). Based on the near-field and effective refractive index, nanocavities comparable to a quarter and three-quarter wavelength proved to be optimal. Based on the geometry shown in the near-field figures, the NCTAI-SNSPD deflectors are so wide that the trench width approaches zero, i.e., it almost adopts NCAI-SNSPD geometry (Figure S7c). This explains the well-defined similarity between the optical responses of the two optimized compositions. The configuration with shorter nanocavities results in larger absorptance.

The two SNSPD compositions show very similar characteristics. Examining the BSCCO absorptance dispersion graph, it can be seen that a plasmonic mode propagates due to first-order lattice coupling; this band folds back from the boundary of the first Brillouin zone (Figure S8). This plasmonic mode appears similarly in all compositions and configurations, proving that in all cases, tuning this coupled mode to 1550 nm results in the highest absorptance. The essential difference between the configurations is that the resonance peak is narrower with the longer nanocavity, in accordance with its higher order nature and higher Q-factor. At the boundary of the first Brillouin zone and in the second zone, increased absorptance is observed over a wide range of frequencies and momenta. This is the result of the plasmonic Brewster angle, which is better localized above the ω-k parameter plane in the longer nanocavity configuration.

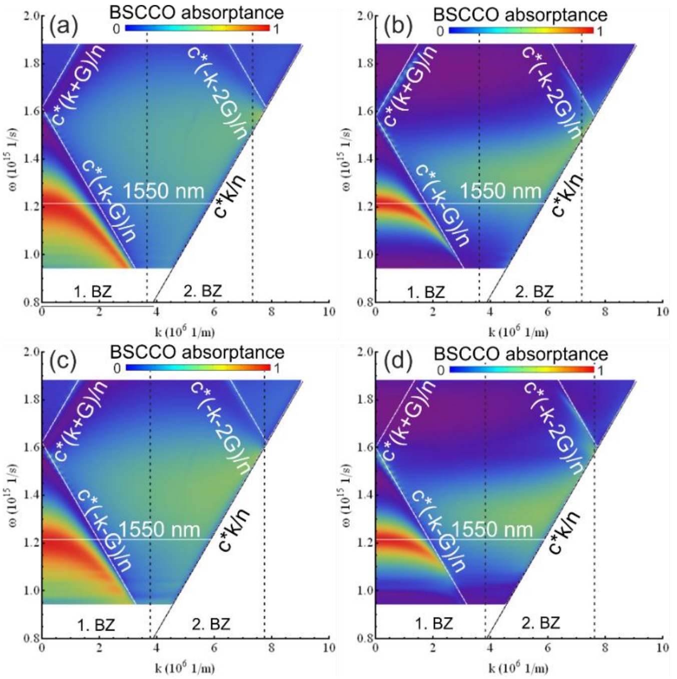

Figure S8. Dispersion diagram of BSCCO absorptance of (a) NCAI-SNSPD in $\lambda/(4 \times n_{eff})$ mode, (b) NCAI-SNSPD in $3 \times \lambda/(4 \times n_{eff})$ mode, (c) NCTAI-SNSPD in $\lambda/(4 \times n_{eff})$ mode and (d) NCTAI-SNSPD in $3 \times \lambda/(4 \times n_{eff})$ cavity resonant mode.

optical response (1550 nm, 0°)	NCAI		NCTAI	
	$\lambda/(4\cdot n_{eff})$	$3\times\lambda/(4\times n_{eff})$	$\lambda/(4\times n_{eff})$	$3\times\lambda/(4\times n_{eff})$
BSCCO absorptance	0.867	0.798	0.844	0.783
Au absorptance	0.090	0.160	0.110	0.176
reflectance	0.002	0.001	0.007	0.009
max. near-field	3.4 (10.2)	3.3 (9.9)	3.8 (10.3)	3.3 (9.4)
n_{eff} (1550 nm)	4	2.3	3.9	2.1
$\lambda/(4\times n_{eff})$ (nm)	96.9	171.4	98.6	181.0
$3\times\lambda/(4\times n_{eff})$ (nm)	290.6	514.2	295.8	543.1
λ/n_{eff} (nm)	387.5	685.5	394.4	724.1
h_{HSQ} (nm)	140.3	621.3	143.6	629.5
total cavity height (nm)	170.3	651.3	173.6	659.5
period (nm)	803.7	822.7	801.2	811.6
cavity width (nm)	246.0	241.8	185.4	231.5
fill factor	30.6%	29.4%	23.1%	28.5%
PBA (°)	72.0	72.7	76.5	70.4

Table S5. Optical response of optimized SNSPD compositions at 1550 nm and perpendicular incidence of 30 nm thick BSCCO wire. The calculated effective refractive index and nanocavity height are also presented and the matched lengths are indicated with a green background. PBA—calculated plasmonic Brewster angle.

Greater absorptance can be achieved with quarter-wavelength nanocavity configurations and among those with the NCAI-SNSPD compositions (Table S5). The highest absorptance is thus 0.867 in the quarter-wavelength NCAI-SNSPD configuration, which is only slightly larger than the 0.844 of the quarter-wavelength NCTAI-SNSPD. The absorptance in the three-quarter-wavelength nanocavity length cases is 0.798 and 0.783 in the NCAI-SNSPD and NCTAI-SNSPD compositions, respectively. The advantage of NCTAI-SNSPD is that it achieves slightly lower absorptance with a smaller fill factor.

The advantage of the 30 nm thick BSCCO wire is that the absorptance can be significantly increased by more than +0.1 compared to the 15 nm thick wire.

Sensitivity study

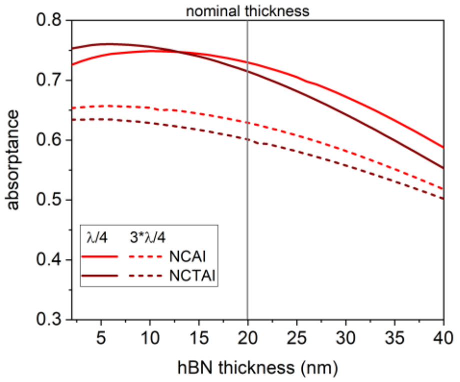

Figure S9. BSCCO absorptance with varied hBN layer thickness. BSCCO thickness = 15 nm.

By varying the hBN thickness, the effect of geometrical uncertainty in the encapsulation layer was assessed. The simulations show that the BSCCO absorptance decreases with increasing hBN thickness. This trend can be attributed to the larger separation between the absorbing BSCCO layer and the nanocavity entrance, which reduces the near-field enhancement experienced by the superconducting layer. In the worst-case scenario considered here, increasing the hBN thickness by 20 nm reduces the BSCCO absorptance from 0.71 to 0.55, corresponding to an approximately 23% decrease.

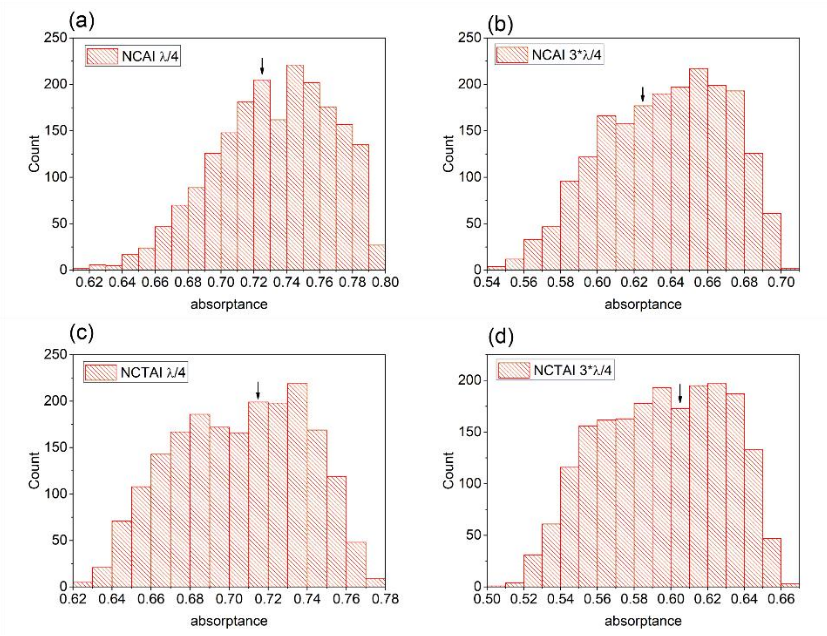

Figure S10. Histogram of BSCCO absorptance values calculated for the optimized geometry with material permittivities varied by ±20%. Arrow indicates the bar corresponding to the values presented in the manuscript.

To assess the effect of material-parameter uncertainty, the nominal optical parameters of BSCCO, hBN, and reBSCCO were varied within a ±20% interval. This sensitivity analysis was performed using Monte Carlo simulations, with 2000 samples generated for each investigated case. Based on the obtained distributions, the BSCCO absorptance shows only moderate sensitivity to the assumed material parameters. Even in the least favorable case, less than 5% of the sampled configurations exhibited an absorptance decrease larger than 10%.

The robustness of the optimized geometry was further evaluated in the representative reference case of NCAI-SNSPD with a $\lambda/(4 \times n_{eff})$ cavity length by allowing random deviations of the geometrical parameters around their nominal optimum values (Figure S11). For a ±5% geometrical-parameter variation, which is commensurate with the uncertainty of the state-of-the-art lithography methods, 90% of the 1000 sampled configurations exhibited an absorptance decrease of less than 14%. When the allowed variation was increased to ±10%, the corresponding decrease remained below 41% for 90% of the samples. In contrast, for a ±20% variation, the absorptance became strongly degraded in some cases, dropping to less than 8% of the optimum value. However, the latter two uncertainties are far below the reproducibility of lithography methods.

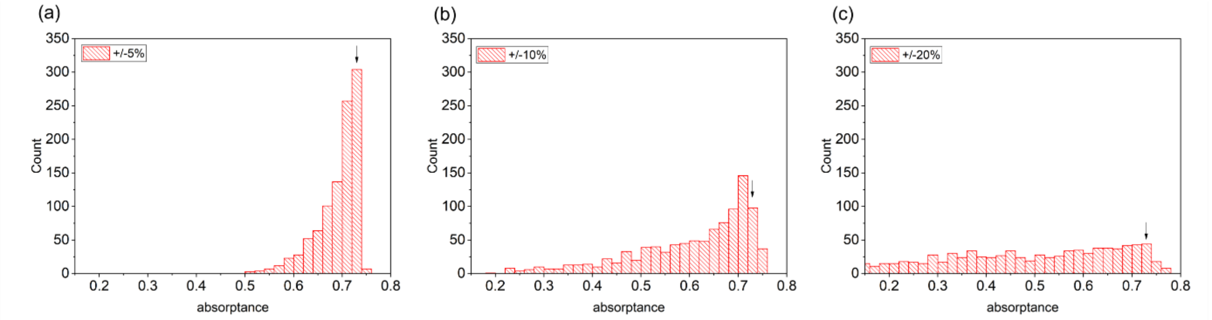

Figure S11. Histogram of BSCCO absorptance values calculated for the optimized geometry with geometric parameters varied by (a) realistic ±5% value and overestimated (b) ±10% and (c) ±20% values. Arrow indicates the bar corresponding to the values presented in the manuscript.

Finally, to verify that the calculated absorptance values are not governed by the numerical discretization, a mesh-convergence test was performed for the optimized geometries. The optical response was recalculated using progressively refined meshes, while keeping all geometrical parameters unchanged. The mesh was refined using a mesh-size multiplier, (s), which scaled the characteristic mesh size in each domain according to *mesh size* × s. The BSCCO absorptance obtained with the final mesh differed from that of the succeeding finer mesh by less than 0.2%, confirming that the numerical models are converged (Figure S12).

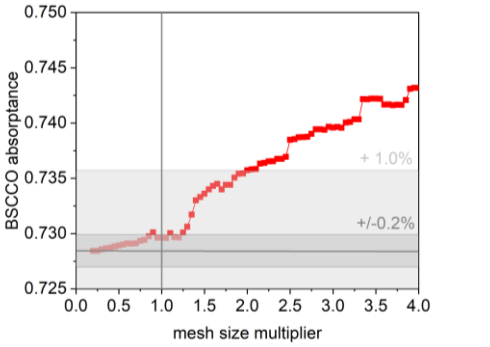

Figure S12. Convergence study in the case of NCAI-SNSPD with $\lambda/(4 \times n_{eff})$ cavity length. The mesh size multiplier denotes a factor which multiplies the used mesh size of 5 nm.

References

[S1] Romanowsky, M. B., Capasso, F. Orientation-dependent Casimir force arising from highly anisotropic crystals: Application to $Bi_2Sr_2CaCu_2O_{8+\delta}$. *Phys. Rev. A* **8**, 042110 (2008).

[S2] Yuan, Z.et al. Extremely Confined Acoustic Phonon Polaritons in Monolayer-hBN/Metal Heterostructures for Strong Light–Matter Interactions. *ACS Photonics*, **7**, 2610–2617 (2009).
